# Weighted Measurement Fusion Particle Filter for Nonlinear Systems with Correlated Noises

**DOI:** 10.3390/s18103242

**Published:** 2018-09-26

**Authors:** Ke Wei Zhang, Gang Hao, Shu Li Sun

**Affiliations:** School of Electronic Engineering, Heilongjiang University, Harbin 150080, China; m18845031131@163.com

**Keywords:** nonlinear system, Taylor series expansion, particle filter, weighted measurement fusion, correlated noises

## Abstract

The multi-sensor information fusion particle filter (PF) has been put forward for nonlinear systems with correlated noises. The proposed algorithm uses the Taylor series expansion method, which makes the nonlinear measurement functions have a linear relationship by the intermediary function. A weighted measurement fusion PF (WMF-PF) was put forward for systems with correlated noises by applying the full rank decomposition and the weighted least square theory. Compared with the augmented optimal centralized fusion particle filter (CF-PF), it could greatly reduce the amount of calculation. Moreover, it showed asymptotic optimality as the Taylor series expansion increased. The simulation examples illustrate the effectiveness and correctness of the proposed algorithm.

## 1. Introduction

In the last few years, filtering algorithms for nonlinear systems have attracted a great deal of attention from many scholars due to their wide applications such as guidance, signal processing, aircraft attitude estimation, trajectory tracking, and so on [1,2,3,4,5,6,7,8]. Due to the low accuracy and the poor fault tolerance of single-sensor systems, multi-sensor information fusion technologies are gaining more and more attention from scholars and have been widely used due to their high accuracy and reliability. This paper proposes a multi-sensor information fusion filter for nonlinear systems with correlated noise.

Nonlinear systems have many filtering methods including the extended Kalman filter (EKF) [9,10], unscented Kalman filter (UKF) [11,12], cubature Kalman filter (CKF) [13], Sequence Monte Carlo (SMC) [14,15], Markov Chain Monte Carlo (MCMC) [16], particle filter (PF) [17,18,19,20,21], and so on. As PF does not demand the system noises to be Gaussian, it can be applied to more situations. In practice, the conditions of noise independence are difficult to meet due to the changes in the internal and external environment. When the measurement noises and the system noises are correlated, the classical filters (EKF, UKF, CKF, PF) will be biased or even divergent. Based on the Bayesian estimation framework, there are two fundamental methods for the estimation problem with correlated noise. One is the de-correlation method [22,23,24,25,26], which can convert the correlated noises into uncorrelated ones. Its advantage is that the algorithm is simple in structure and implementation. The other is to redesign the new filters by improving the existing algorithm framework [27,28,29,30,31]. Its advantage is that the algorithm generally has less computational cost than the de-correlation method, and its disadvantage is that the algorithm does not have a general structure and is difficult to design. Taking into account the universality, the de-correlation method was used in this paper.

So far, there are two main kinds of fusion methods: the centralized fusion (CF) algorithm and distributed information fusion algorithms. The CF algorithm spreads all sensor information and outputs into a fusion state estimate. The CF algorithm is globally optimal under certain performance criteria when all sensors work well, because there is no information lost [32]. However, the disadvantages of the CF algorithm include the expensive computational cost and poor fault tolerance [32,33]. The distributed fusion algorithms send local state estimates to the fusion center and perform weight estimation according to a certain fusion criterion [34,35,36,37]. Distributed fusion algorithms have a lower computational cost and better fault tolerance than the centralized fusion algorithm. However, the estimated accuracy is globally suboptimal and locally optimal. In order to ensure the accuracy of the estimation, a kind of centralized fusion method, the weighted measurement fusion (WMF) algorithms, was adopted in this paper. Unlike the traditional centralized fusion method, the WMF algorithms fuse the measurements according to certain criterion, then the dimensional measurement is used in the filter for estimating. The WMF is a kind of data compression method and widely used in the occasions of mass data such as the sensor network and so on [38,39,40,41,42]. The WMF algorithms require that the measurement noises and the system noises are independent, and the filters [41] can deal with the filtering problem with correlated noise by the Lagrange multiply method. However, both of them can only be used in linear systems.

In this paper, a WMF algorithm was proposed for nonlinear systems with correlated noises by using the weighted least squares (WLS) method and Taylor series expansion. It can compress a high-dimensional measurement into a low-dimensional measurement for nonlinear measurements. Furthermore, a weighted measurement fusion particle filter (WMF-PF) was presented by combining it with PF. The proposed filter could solve the fusion estimation problem for multi-sensor nonlinear systems with correlated measurement noises and the system noises. The proposed WMF-PF had an approximate accuracy and a reduced calculation when compared with the centralized fusion particle filter.

The rest of the paper is organized as follows. The de-correlation and weighted measurement fusion algorithms are introduced in Section 2. Combined with PF, a weighted measurement fusion particle filter (WMF-PF) algorithm is introduced in Section 3. The simulation analysis is given in Section 4. The conclusions are summarized in Section 5.

## 2. Problem Formulation

Consider the following nonlinear multiple sensor systems with correlated noises:

(1) x(k+1)=f(x(k),k)+Γw(k) 

(2) $$y_{j}(k)=h^{\left(j\right)}(x(k),k)+v^{\left(j\right)}(k),\;j=1,2,\cdots ,L\;$$

where k is the discrete time; $x(k)\in R^{n}$ is the system state vector at time k; $y_{j}(t)\in R^{m_{j}}$ is the measurement vector of the *j*th sensor; $w(k)∼p_{\omega }(\cdot )$ is the system noise and $v_{j}(k)∼p_{v_{j}}(\cdot )$ is the measurement noise of the *j*th sensor; $f(\cdot ,\cdot )\in R^{n}$ and $h_{j}(\cdot ,\cdot )\in R^{m_{j}}$ are the nonlinear state and measurement functions, respectively; and $\Gamma \in R^{n\times p}$ is the noise input matrix. L is the number of sensors. w(k) and $v_{j}(k)$ are correlated white noises with zero mean and covariance $Q_{w}$ and $R_{ji}$, i.e.,

(3) $$E\left\{\begin{array}{cc} \left[\begin{array}{c} w(t)\\ v_{j}(t) \end{array}\right] & \left[\begin{array}{cc} w^{T}(k) & {v_{i}}^{T}(k) \end{array}\right] \end{array}\right\}=\left[\begin{array}{cc} Q_{w} & S_{i}\\ S_{j}^{T} & R_{ji} \end{array}\right]\delta_{tk}\;$$

where E is defined as a mathematical expectation; the superscript T is defined as the transpose; and $\delta_{tk}$ is the Kronecker-ä function, i.e., $\delta_{tt}=1$, $\delta_{tk}=0(t\neq k)$.

Next, we converted the systems in Equations (1) and (2) into the systems with independent noise.

**Theorem 1.**  *For systems in Equations (1) and (2), the decorrelated centralized measurement fusion systems (CMFS) can be rewritten as:*

(4) $$x(k+1)=\bar{f}(x(k),k)+\bar{w}(k)\;$$

(5) $$y^{\left(C\right)}(k)=h^{\left(C\right)}(x(k),k)+v^{\left(C\right)}(k)\;$$

*where:*

(6) $$\bar{f}(x(k),k)=f(x(k),k)-Mh^{\left(C\right)}(x(k),k)+My^{\left(C\right)}(k)\;$$

(7) $$M=\Gamma S^{\left(C\right)}{\left(R^{\left(C\right)}\right)}^{-1}\;$$

(8) $$\bar{w}(k)=\Gamma w(k)-Mv^{\left(C\right)}(k)\;$$

(9) $$y^{\left(C\right)}(k)={\left[y_{1}^{T}(k),y_{2}^{T}(k),\cdots ,y_{L}^{T}(k)\right]}^{T}\;$$

(10) $$h^{\left(C\right)}(x(k),k)={\left[h_{1}^{T}(x(k),k),h_{2}^{T}(x(k),k),\cdots ,h_{L}^{T}(x(k),k)\right]}^{T}\;$$

(11) $$v^{\left(C\right)}(k)={\left[v_{1}^{T}(k),v_{2}^{T}(k),\cdots ,v_{L}^{T}(k)\right]}^{T}\;$$

*and the covariance matrix of* $v^{\left(C\right)}(k)$ *is given as:*

(12) $$R^{\left(C\right)}={\left(R_{ij}\right)}_{m\times m},\;m=\sum_{i=1}^{L}m_{i}\;$$

*The cross-covariance matrix of* w(k) *and* $v^{\left(C\right)}(k)$ *is given as:*

(13) $$S^{\left(C\right)}=[S_{1},S_{2},\cdots ,S_{L}]\;$$

*and the system noise statistical property after the transformation is as follows:*

(14) $$E[\bar{w}(k)]=0\;$$

(15) $$E[\bar{w}(k){\bar{w}}^{T}(k)]=\Gamma [Q_{w}-S^{\left(C\right)}{\left(R^{\left(C\right)}\right)}^{-1}{\left(S^{\left(C\right)}\right)}^{T}]\Gamma^{T}\;$$

**Proof.**  From Equation (2), merging the L measurement equations, Equations (5) and (9)–(13) are obtained. Equation (1) can be transformed via Equations (1) and (2):

(16) $$x(k+1)=f(x(k),k)+\Gamma w(k)+M(y^{\left(C\right)}(k)-h^{\left(C\right)}(x(k),k)-v^{\left(C\right)}(k))\;$$

Then, we obtain Equations (4), (6) and (8), and the measurement noises and the system noises are uncorrelated in Equations (4) and (5), i.e.,

(17) $$E[\bar{w}(k)v^{(C)T}(k)]=0\;$$

We can obtain the M as Equation (7). □

For multi-sensor systems, the centralized fusion method above-mentioned will have a high computational cost due to the high-dimensional augmented measurements. Therefore, many scholars have put forward a weighted measurement fusion method that can compress an augmented high dimensional measurement into a low dimensional measurement. However, many existing results refer to linear systems. In the following, we propose a weighted measurement fusion algorithm to solve the nonlinear systems with correlated noises.

**Theorem 2.**  *For the systems in Equations (4) and (5), if the measurement functions*$h^{\left(j\right)}(x(k),k)$*,*j=1,2,⋯,L*have linear relationships, i.e.,*$\exists h(x(k),k)\in {ℜ}^{p}$*satisfies*$h_{j}(x(k),k)=H_{j}(k)h(x(k),k)$*, with matrix*$H_{j}(k)\in {ℜ}^{m_{j}\times p}$. *The reduced dimensional measurement function of the weighted measurement fusion system (WMFS) is as follows:*

(18) $$y^{\left(I\right)}(k)=H^{\left(I\right)}(k)h(x(k),k)+v^{\left(I\right)}(k)\;$$

*where:*

(19) $$y^{\left(I\right)}(k)={\left[K^{T}(k){\left(R^{\left(C\right)}\right)}^{-1}K(k)\right]}^{-1}K^{T}(k){\left(R^{\left(C\right)}\right)}^{-1}y^{\left(C\right)}(k)\;$$

(20) $$v^{\left(I\right)}(k)={\left[K^{T}(k){\left(R^{\left(C\right)}\right)}^{-1}K(k)\right]}^{-1}K^{T}(k){\left(R^{\left(C\right)}\right)}^{-1}v^{\left(C\right)}(k)\;$$

K(k) *is a full-column rank matrix and* $H^{\left(I\right)}(k)$ *is a full-row rank matrix, which are the full rank decomposition matrices of matrix* $H^{\left(C\right)}(k)\in {ℜ}^{m\times p}$ *, i.e.,*

(21) $$H^{\left(C\right)}(k)={\left[H_{1}^{T}(k),H_{2}^{T}(k),\cdots ,H_{L}^{T}(k)\right]}^{T}=K(k)H^{\left(I\right)}(k)\;$$

*The covariance matrix of* $v^{\left(I\right)}(k)$ *is computed as:*

(22) $$R^{\left(I\right)}(k)={\left[K^{T}(k)R^{(C)-1}K(k)\right]}^{-1}\;$$

**Proof.**  Using $h_{j}(x(k),k)=H_{j}(k)h(x(k),k)$ and substituting Equation (21) into Equation (5), we have:

(23) $$y^{\left(C\right)}(k)=K(k)H^{\left(I\right)}(k)h(x(k),k)+v^{\left(C\right)}(k)\;$$

As $K^{T}(k)R^{(C)-1}K(k)$ is nonsingular, the optimal Gauss-Markov estimate of $H^{\left(I\right)}(k)h(x(k),k)$ can be obtained as Equations (18)–(22) by using the WLS method. □

For some nonlinear systems, the intermediary function h(x(k),k) is difficult to obtain. In the following, we present a polynomial intermediary function obtained by the Taylor series expansion method that can overcome the constraints of Theorem 2, so that the WMFS can be obtained via Theorem 2.

**Theorem 3.**  *The approximate measurement equation of WMFS for the system in Equations (4) and (5) is given as:*

(24) $${\tilde{y}}^{\left(I\right)}(k)={\tilde{H}}^{\left(I\right)}(k)\tilde{h}(x(k),k)+{\tilde{v}}^{\left(I\right)}(k)\;$$

*where:*

(25) $${\tilde{y}}^{\left(I\right)}(k)={\left[{\tilde{K}}^{T}(k){\left(R^{\left(C\right)}\right)}^{-1}\tilde{K}(k)\right]}^{-1}{\tilde{K}}^{T}(k)R^{(C)-1}y^{\left(C\right)}(k)\;$$

(26) $${\tilde{v}}^{\left(I\right)}(k)={\left[{\tilde{K}}^{T}(k){\left(R^{\left(C\right)}\right)}^{-1}\tilde{K}(k)\right]}^{-1}{\tilde{K}}^{T}(k){\left(R^{\left(C\right)}\right)}^{-1}v^{\left(C\right)}(k)\;$$

*The covariance matrix of*${\tilde{v}}^{\left(I\right)}(k)$*is*${\tilde{R}}^{\left(I\right)}(k)={\left[{\tilde{K}}^{T}(k)R^{(C)-1}\tilde{K}(k)\right]}^{-1}$*, and*${\tilde{H}}^{\left(I\right)}(k)$*and*${\tilde{K}}^{T}(k)$*are the full rank decomposition matrices of*${\tilde{H}}^{\left(C\right)}(k)$*as Equation (21), where:*

(27) $${\tilde{H}}^{\left(C\right)}(k)={\left[\begin{array}{ccccc} D^{(C)0} & D^{(C)1} & D^{(C)2} & \cdots & D^{(C)\gamma } \end{array}\right]|}_{x=\hat{x}}\;$$

(28) $$\tilde{h}(x(k),k)={\left[\begin{array}{ccccc} 1 & {\left(\Delta x\right)}^{T} & {\left({\left(\Delta x\right)}^{2}\right)}^{T} & \cdots & {\left({\left(\Delta x\right)}^{\gamma }\right)}^{T} \end{array}\right]}^{T}\;$$

(29) $$D^{(C)l}=\frac{1}{l!}\frac{\partial^{l}h^{\left(C\right)}(x,k)}{\partial x^{T}},l=1,\cdots ,\gamma \;$$

γ*is the order of Taylor series expansion, and defining:*

(30) $$\Delta x=x-\hat{x}\;$$

(31) $${\left(\Delta x\right)}^{l}={\left[\begin{array}{ccc} \prod_{i=1}^{n}\Delta x_{i}^{l_{i}}, & \cdots , & \prod_{j=1}^{n}\Delta x_{j}^{l_{j}} \end{array}\right]}^{T},\;\sum_{k=1}^{n}l_{k}=l\;$$

(32) $$D^{(C)l}{|}_{x=\hat{x}}{\left(\Delta x\right)}^{l}=[\begin{array}{c} \sum \frac{\partial^{l}h_{1}^{\left(C\right)}(x,k)}{\partial x_{1}^{l_{1}}x_{2}^{l_{2}}\cdots x_{n}^{l_{n}}}(\Delta x_{1}^{l_{1}}\cdot \Delta x_{2}^{l_{2}}\cdot \cdots \cdot \Delta x_{n}^{l_{n}})\\ \sum \frac{\partial^{l}h_{2}^{\left(C\right)}(x,k)}{\partial x_{1}^{l_{1}}x_{2}^{l_{2}}\cdots x_{n}^{l_{n}}}(\Delta x_{1}^{l_{1}}\cdot \Delta x_{2}^{l_{2}}\cdot \cdots \cdot \Delta x_{n}^{l_{n}})\\ \vdots \\ \sum \frac{\partial^{l}h_{m}^{\left(C\right)}(x,k)}{\partial x_{1}^{l_{1}}x_{2}^{l_{2}}...x_{n}^{l_{n}}}(\Delta x_{1}^{l_{1}}\cdot \Delta x_{2}^{l_{2}}\cdot \cdots \cdot \Delta x_{n}^{l_{n}}) \end{array}],\;\sum_{i=1}^{n}l_{i}=l\;$$

*where*$h_{i}^{\left(C\right)}(x,k),\;i=1,\cdots ,m$*is ith the component of*$h^{\left(C\right)}(x(k),k)$.

**Proof.**  Using the Taylor series expansion method, the approximation of $y^{\left(C\right)}(k)$ in Equation (23) can be written as:

(33) $${\tilde{y}}^{\left(C\right)}(k)={h^{\left(C\right)}(x,k)|}_{x=\hat{x}}+{\frac{\partial h^{\left(C\right)}(x,k)}{\partial x^{T}}|}_{x=\hat{x}}\Delta x+\frac{1}{2!}{\frac{\partial^{2}h^{\left(C\right)}(x,k)}{\partial x^{T}}|}_{x=\hat{x}}{\left(\Delta x\right)}^{2}+\cdots \;+\frac{1}{\gamma !}{\frac{\partial^{\gamma }h^{\left(C\right)}(x,k)}{\partial x^{T}}|}_{x=\hat{x}}{\left(\Delta x\right)}^{\gamma }+v^{\left(C\right)}(k)\;=[\begin{array}{cccc} h^{\left(C\right)}(x,k), & \frac{\partial h^{\left(C\right)}(x,k)}{\partial x^{T}}, & \frac{1}{2!}\frac{\partial^{2}h^{\left(C\right)}(x,k)}{\partial x^{T}}, & \cdots , \end{array}{\frac{1}{\gamma !}\frac{\partial^{\gamma }h^{\left(C\right)}(x,k)}{\partial x^{T}}]|}_{x=\hat{x}}{\left[\begin{array}{cccc} 1 & \Delta x & \cdots & {\left(\Delta x\right)}^{\gamma } \end{array}\right]}^{T}+v^{\left(C\right)}(k)\;$$

Let $\tilde{h}(x(k),k)$ be Equation (28) and ${\tilde{H}}^{\left(C\right)}(k)$ be Equation (27), then Equations (24)–(26) can be obtained by Theorem 2.

By increasing the number of sensors, the dimension of the measurement equation of CMFS will be large and it will cause an expensive computational cost. However, the dimension of the measurement equation of WMFS will be fixed when the order of the Taylor series expansion γ is fixed. For example, if the dimensions of the system and measurement functions are both 1 and there are 10 sensors, the dimension of the measurement equation of CMFS is 10, but the dimension of the measurement equation of WMFS is 3 when we choose γ=2. □

## 3. Weighted Measurement Fusion Particle Filter (WMF-PF) Algorithm

(1) Initialization:

(34) $${\hat{x}}^{\left(I)(i\right)}(0|0)∼p_{x_{0}}(x_{0}),\;i=1,\cdots ,N_{s}\;$$

(2) State prediction particles:

(35) $${\hat{x}}^{\left(I)(i\right)}(k|k-1)=f({\hat{x}}^{\left(I)(i\right)}(k-1|k-1),k-1)+\xi^{\left(I)(i\right)}(k-1)\;$$

where $\xi^{\left(I)(i\right)}(k-1)$ is the random number with the same distribution of the process noise w(k), and the measurement prediction particles:

(36) $${\hat{y}}^{\left(I)(i\right)}(k|k-1)={\tilde{H}}^{\left(I\right)}(k)h({\hat{x}}^{\left(I)(i\right)}(k|k-1),k)\;$$

(3) The importance weights:

(37) $$\omega_{k}^{\left(I)(i\right)}=\frac{1}{N_{s}}p({\hat{y}}^{\left(I)(i\right)}(k|k-1)|{\hat{x}}^{\left(I)(i\right)}(k|k-1))\;$$

that is:

(38) $$\omega_{k}^{\left(I)(i\right)}=\frac{1}{N_{s}}p_{v_{k}^{\left(I\right)}}({\tilde{y}}^{\left(I\right)}(k)-{\hat{y}}^{\left(I)(i\right)}(k|k-1))\;$$

where ${\tilde{y}}^{\left(I\right)}(k)$ is computed by Equation (25) and ${\bar{\omega }}_{k}^{\left(I)(i\right)}$ is given as:

(39) $${\bar{\omega }}_{k}^{\left(I)(i\right)}=\frac{\omega_{k}^{\left(I)(i\right)}}{\sum_{i=1}^{N}\omega_{k}^{\left(I)(i\right)}}\;$$

(4) Filter and filter error variance matrix:

(40) $${\hat{x}}^{\left(I\right)}(k|k)=\sum_{i=1}^{N_{s}}{\bar{\omega }}_{k}^{\left(I)(i\right)}{\hat{x}}^{\left(I)(i\right)}(k|k-1)\;$$

(41) $$P^{\left(I\right)}(k|k)\approx \sum_{i=1}^{N_{s}}{\bar{\omega }}_{k}^{\left(I)(i\right)}{\left({\hat{x}}^{\left(I)(i\right)}(k|k-1)-{\hat{x}}^{\left(I)(i\right)}(k|k)\right)}^{2}\;$$

(5) Resampling:

(42) $$u_{i}=\frac{(i-1)+r}{N},\;r∼U[0,1],\;i=1,\cdots ,N_{s}\;$$

If $\sum_{j=1}^{m-1}{\bar{\omega }}_{k}^{\left(I)(j\right)}<u_{i}\leq \sum_{j=1}^{m}{\bar{\omega }}_{k}^{\left(I)(j\right)}$, we directly copied m particles as the resampling particles ${\hat{x}}^{\left(I)(i\right)}(k|k)$ directly.

We then turn to step (2) and iterated.

**Figure 1:**
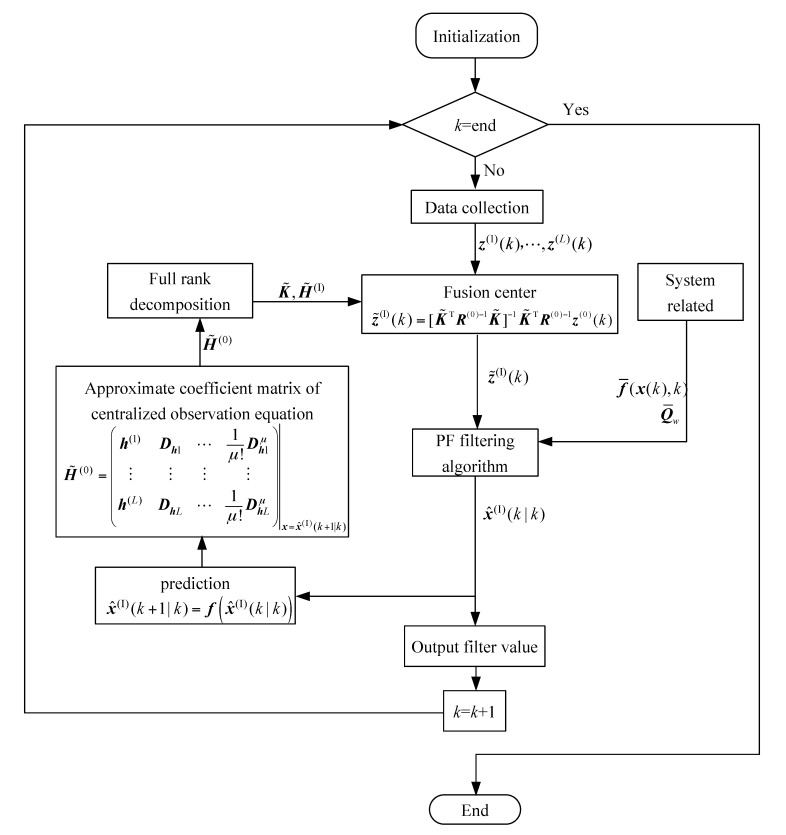
Flow chart of WMF-PF algorithm with correlated noises.

The flow chart of the noise-related WMF-PF algorithm is shown in Figure 1.

## 4. Simulation Example

Let us consider a 4-sensor nonlinear system [43]:

(43) $$x(k)=0.5x(k-1)+25x(k-1)/\left(1+x{\left(k-1\right)}^{2}\right)+8\cos(1.2(k-1))+w(k)\;$$

(44) $$z^{\left(j\right)}(k)=h^{\left(j\right)}\left(x(k),k\right)+v^{\left(j\right)}(k),\;j=1,2,3,4\;$$

$w(k)=\alpha \xi (k)+w_{e}(k)$, $v^{\left(j\right)}(k)=\beta^{\left(j\right)}\xi (k)+v_{e}^{\left(j\right)}(k)$, where ξ(k) is the background noise and $\xi (k)\sim N(0,\;\sigma_{\xi }^{2})$, $w_{e}(k)\sim N(0,\;\sigma_{we}^{2})$, $w_{v}^{\left(j\right)}(k)\sim N(0,\;\sigma_{vj}^{2})$, so

(45) $$S_{j}=\alpha \sigma_{\xi }^{2}{\left(\beta^{\left(j\right)}\right)}^{T},\;R_{ji}=\beta^{\left(j\right)}\sigma_{\xi }^{2}{\left(\beta^{\left(i\right)}\right)}^{T}.$$

In the simulation, we set $\sigma_{\xi }^{2}=0.4^{2}$, $\sigma_{we}^{2}=0.5^{2}$, $\sigma_{v1}^{2}=0.6^{2}$, $\sigma_{v2}^{2}=0.7^{2}$, $\sigma_{v3}^{2}=0.8^{2}$, and $\sigma_{v4}^{2}=0.9^{2}$ as well as α=0.8, $\beta^{\left(1\right)}=1.5$, $\beta^{\left(2\right)}=1.2$, $\beta^{\left(3\right)}=1.4$, and $\beta^{\left(4\right)}=1.3$. 

We selected single-valued functions within the range of x(k), taking into account observability:

(46) h(1)(x(k),k)=x(k); h(2)(x(k),k)=x(k)3/30;h(3)(x(k),k)=10exp(x(k)/10); h(4)(x(k),k)=10sin(π40x(k)),

and the initial value is x(0)=0.

**Figure 2:**
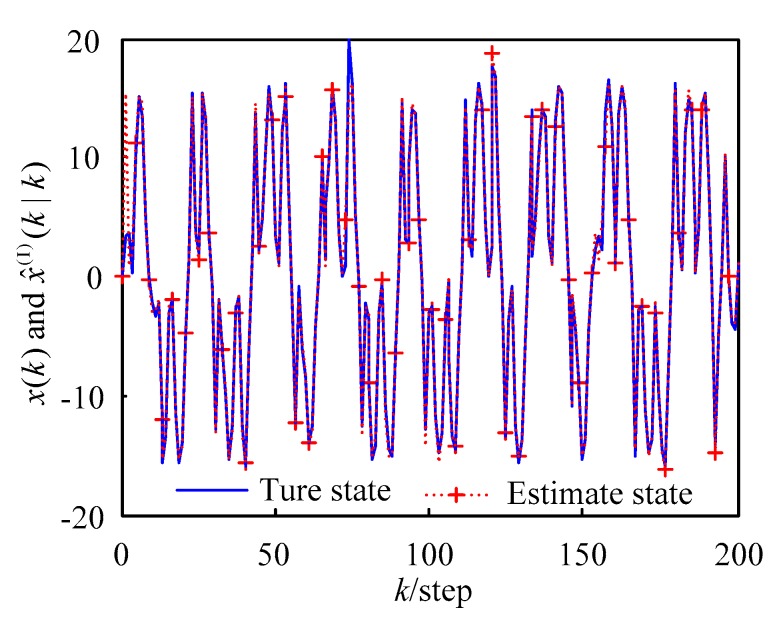
Curves of the true values and estimates using the WMF-PF1.

**Figure 3:**
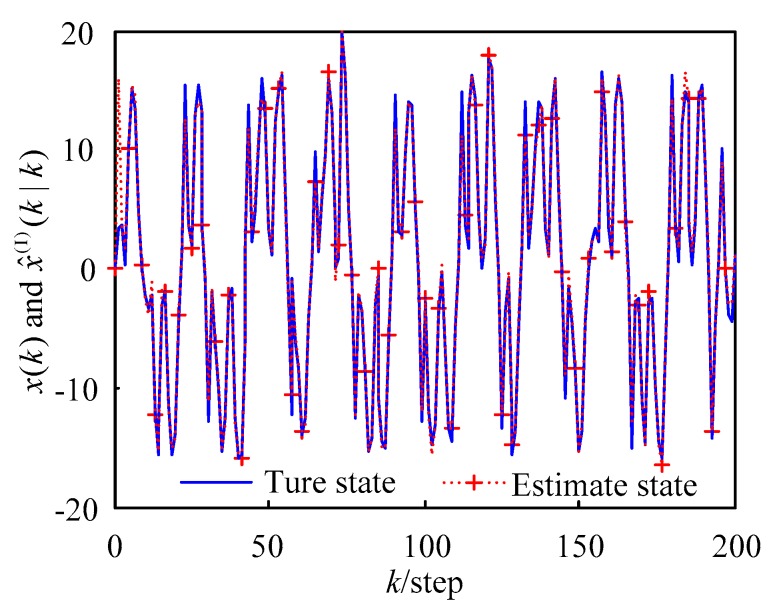
Curves of the true values and estimates using the WMF-PF2.

The first-order Taylor series expansion (WMF-PF1) tracking curve is shown in Figure 2. It can be seen in the figure that the tracking effect was good. The second-order Taylor series expansion (WMF-PF2) tracking curve is shown in Figure 3 where it can be seen that the tracking effect was good.

**Figure 4:**
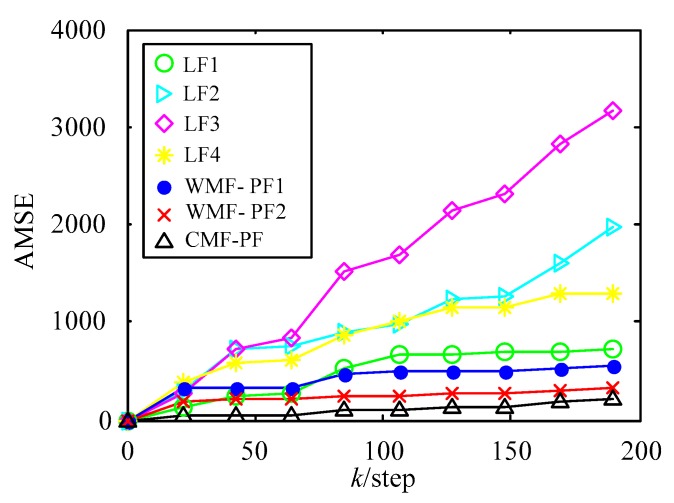
AMSE curves of local PFs, WMF-PF1, WMF-PF2, and CMF-PF.

The estimation performance criterion is the accumulated mean square error (AMSE) [43]:

(47) $$\mathrm{AMSE}(k)=\sum_{t=0}^{k}\frac{1}{N}\sum_{j=1}^{N}{\left[x_{j}(t)-{\hat{x}}_{j}(t|t)\right]}^{2},\;N=50\;$$

where ${\hat{x}}_{j}(t|t)$ is the *j*th-time Monte Carlo experiment at time *t*. The AMSE curves of local PFs (LF1–LF4), the curve of WMF-PF1, the curve of WMF-PF2, and the curve of CF-PF are shown in Figure 4 with 50 Monte Carlo experiments. From Figure 2 and Figure 3 it is evident that all of the fusion filters had better accuracy than the local PFs. Moreover, WMF-PF2 had better accuracy than WMF-PF1, but was worse than CF-PF.

Using WMF-PF2 as an example to analyze the computational burden, the augmented measurement function of CF-PF will be four dimensions. However, for WMF-PF2, the measurement function is three dimensional, so the computational burden of CF-PF will be more expensive than WMF-PF. Moreover, as the number of sensors increases, the dimension of the measurement function of CF-PF will consistently rise, but the measurement function’s dimension of WMF-PF2 will be fixed at three when the order is γ=2.

It can be seen from Figure 3 that the estimation accuracy from high to low was the centralized fusion PF (CF-PF), weighted measurement fusion based on the second-order Taylor series expansion PF algorithm (WMF-PF2), weighted measurement fusion based on the first-order Taylor series expansion PF algorithm (WMF-PF1), and the four local PF estimation algorithms (LF1–LF4).

From the simulation results in Figure 2, Figure 3 and Figure 4, the effectiveness of the de-correlated weighted measurement fusion PF filtering algorithm proposed in this paper can be fully explained.

## 5. Conclusions

For a nonlinear multi-sensor system with correlated noise, the system noise and the measurement noise are correlated at the same time stamp. First, by using a de-correlation method, a nonlinear multi-sensor system with correlated noise was transformed into a nonlinear system with independent Gaussian noise. Then, based on the Taylor series approximation method combined with PF algorithms, the weighted measurement fusion PF algorithm was designed. In fact, the proposed fusion algorithm could be combined with many nonlinear filter algorithms (UKF, PF, CKF, etc.). The proposed filter can be used for multi-sensor discrete-time nonlinear systems with correlated measurement noises and system noises. This algorithm asymptotically approximated the CF-PF with the increase of the Taylor series, so it was approximately globally optimal. The algorithm solved the fusion estimation problem of nonlinear systems, and had an asymptotical accuracy. The fusion algorithm was much less computationally intensive than the centralized fusion algorithm. From the simulation, we could see that the weighted measurement fusion PF had a very low computational complexity and good real-time performance. The algorithm can be widely applied in sensor networks.
